# Design, Synthesis and Anticancer Evaluation of Fangchinoline Derivatives

**DOI:** 10.3390/molecules22111923

**Published:** 2017-11-08

**Authors:** Yazhou Liu, Bin Xia, Junjie Lan, Shengcao Hu, Lan Huang, Chao Chen, Xueyi Zeng, Huayong Lou, Changhu Lin, Weidong Pan

**Affiliations:** 1 State Key Laboratory of Functions and Applications of Medicinal Plants, Guizhou Medical University, 3491 Baijin Road, Guiyang 550014, China; liuyazi19@gmail.com (Y.L.); m18311511109@163.com (B.X.); lanjunjie2007@163.com (J.L.); husheng0221@gmail.com (S.H.); huanglan0612@163.com (L.H.); cc283818640@163.com (C.C.); xueyizeng@126.com (X.Z.); loouhy@126.com (H.L.); 2 The Key Laboratory of Chemistry for Natural Products of Guizhou Province and Chinese Academy of Sciences, 3491 Baijin Road, Guiyang 550014, China

**Keywords:** fangchinoline, derivatives, anticancer activity, apoptosis

## Abstract

Twenty fangchinoline derivatives were synthesized from the natural product fangchinoline, and their anticancer activities on human breast cancer MDA-MB-231 cell line, human prostate cancer PC3 cell line, human melanoma WM9 cell line and human leukaemia HEL and K562 cell lines were evaluated. The biological result showed that those derivatives exhibited potent activities on inhibiting cancer cell growth, and the structure-activity relationships were investigated. Among them, compound **4g**, which was protected by benzoyl group in 7-phenolic position and nitrified in 14-position, showed impressive inhibition on all 5 cancer cell lines, especially WM9 cell line, with an IC_50_ value of 1.07 µM. Further mechanistic studies demonstrated that compound **4g** may induce cancer cell death by apoptotic means. These research results suggested that compound **4g** could be a lead for the further development toward an anticancer agent against human melanoma WM9 in the future.

## 1. Introduction

Cancer is one the most crucial public health concerns across the world. Based on the estimation of the World Health Organization (WHO), 8.8 million people died from cancer in 2015, occupying 16.7% of all global death [1]. According to the world cancer report, breast cancer has the highest incidence among women (43.3 per 100,000) while prostate cancer has the second-highest among men (31.1 per 100,000) [2]. Melanoma is another aggressive human cancer, the incidence of which has dramatically increased during the past decades, although racial difference varies the risk of developing this kind of cancer [3]. Leukaemia usually begins in bone marrow, and results in large numbers of abnormal white blood cells, which occurs most often in adults older than 55 years and children younger than 15 years [4].

**Figure 1:**
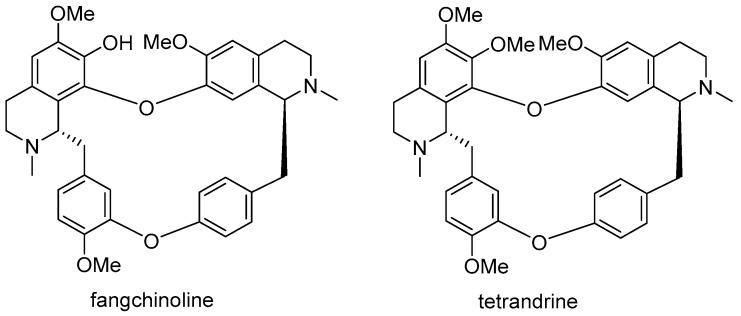
The structure of fangchinoline and tetrandrine.

Chemotherapy remains one of the most important methods for cancer treatment [5,6]. Natural products play an important role in the context of anti-cancer drug discovery. Statistics data indicates that more than 50% of drugs were derived from natural products, either natural origins or derivatives of natural origins [7,8,9]. Fangchinoline and tetrandrine (Figure 1) are two bisbenzylisoquinoline alkaloids isolated from a Chinese traditional herb *Stephaniae tetrandra* S. Moore, which is mainly used as an analgesic and anti-hypertension agent in China [10]. Many bioactivities have been discovered since fangchinoline and tetrandrine were isolated as two of the main active ingredients in *S. tetrandra* [11,12,13,14,15,16,17,18]. Our group has previously reported that fangchinoline could induce autophagy via P53/sestrin2/AMPK signalling in human hepatocellular carcinoma cell lines, HepG2, and PLC/PRF/5 at relatively low concentrations (IC_50_ was approximately 5 µM) [19]. While tetrandrine, which has similar chemical structure, has been reported to arrest the G1/S and G2/M cell cycle and stimulate apoptotic cell death and autophagy via PKC-alpha Inhibition and mTOR-Dependent Mechanisms [16,17,19]. Several derivatives of tetrandrine have been synthesized and evaluated by our team since the unique activity was discovered [20,21]. However, little research about the derivatization and evaluation of fangchinoline has been released.

One of the hallmarks of cancer is the dysregulation of the apoptotic pathways in cells [22,23]. Apoptosis (programmed cell death), often occurs in multicellular organisms to eliminate unnecessary and unwanted cells [24,25]. Many changes occur, including blebbing, cell shrinkage, and nuclear fragmentation, while the apoptosis takes place [26]. Much research has revealed revealed that intracellular proteolytic cascade mediates this kind of cell death, believing that the inhibitor of the apoptosis family of proteins and Bcl-2 family of intracellular proteins are the main intracellular regulators of apoptosis [27]. The intrinsic pathway (also called the mitochondrial pathway) and the extrinsic pathway are the two main pathways of apoptosis [28].

Here, we describe the design and synthesis of fangchinoline derivatives. The biological activities on several cancer cell lines, including human breast cancer MDA-MB-231 cell line, human prostate cancer PC3 cell line, human melanoma WM9 cell line and human leukaemia HEL and K562 cell lines, were evaluated, followed by the proposed structure activity relationships. Moreover, the primary mechanism of action was also investigated.

## 2. Results and Discussion

### 2.1. Chemistry

**Scheme 1:**
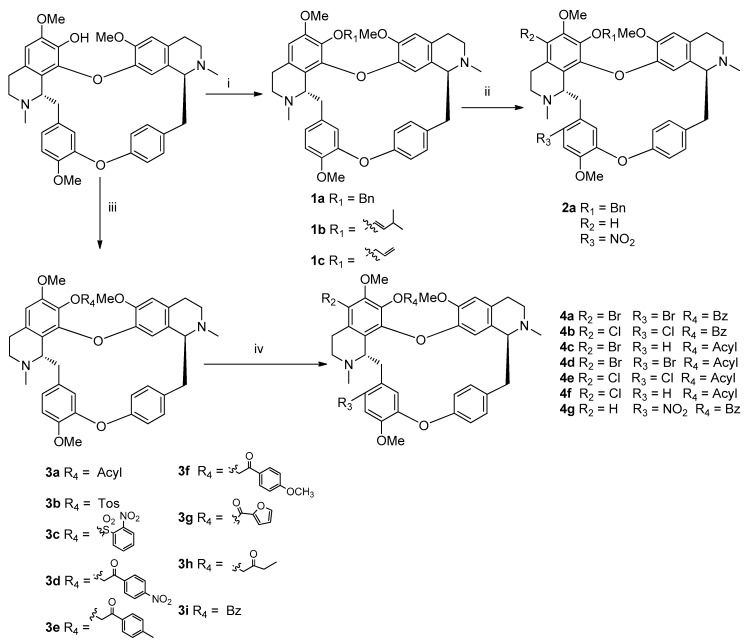
Synthetic route of fangchinoline derivatives **1**–**4**. (i) sodium hydride, halide, r.t.; (ii) HNO_3_, H_2_SO_4_, −10–0 °C; (iii) acyl halide, DMAP, r.t.; (iv) NBS or NCS, TFA, 0 °C–r.t.

Twenty fangchinoline derivatives were designed and synthesized. The derivatization of fangchinoline is summarized in Scheme 1. The strategy of derivatization is to protect 7-phenolic position with different groups then introduce halogens or nitryl on 5- and/or 14-position(s) to form the “first stage” and “second stage” analogues. For the first stage, compounds **1a** to **1c** are the primary ether-protected derivatives, while compounds **3a** to **3i** are protected by acyl groups. Both of these two series of primary analogues were formed through routes I and III respectively. Then, halogens and nitro group were induced to some of those primary analogues as the secondary stage, forming compound **2a** and compounds **4a** to **4g** for the purpose of obtaining improved anticancer activities by routes II and IV.

### 2.2. Biological Evaluation

#### 2.2.1. In Vitro Cytotoxicity Assay

As summarized in Table 1, five cancer cell lines, including MDA-MB-231, PC3, WM9, HEL and K562 cell lines, were induced for testing the anti-cancer activities of those derivatives. The first line anti-cancer drug, vincristine, was treated as positive control. All the test compounds were dissolved in DMSO. The preliminary structure-activity relationship of all compounds against the 5 cancer cell lines was also investigated.

The anticancer activities of those derivatives were obviously enhanced compared to the starting material, fangchinoline. Most anticancer activities of those derivatives were at the same level or exceed the positive control. To be specific, when we compared the activities on HEL cell line, the “first stage” products (**1a** to **1c** and **3a** to **3i**) showed better anticancer activities than the “secondary stage” products. For the “first stage” products, with the inducing of the protecting groups on 7-phenolic hydroxy, both the “ester” compounds (**3a**, **3d** to **3i**) and “ether” compounds (**1a** to **1c**) exhibited better results than the “sulfonate” compounds (**3b** and **3c**), for which the former two series of compounds were the same level as that of vincristine. For the PC3 cell line, the “secondary stage” products showed better inhibition activities than “first stage” products with most of their IC_50_ lower than their starting products. One thing should be mentioned; compounds **3a** and **3i**, which were protected by acetyl and propionyl groups, displayed better activities in “first stage” products with IC_50_ values of 3.01 µM and 2.51 µM, respectively, than those of the “aromatic” esters (**3d** to **3g** and **3i**) and “aromatic” sulfonate (**3b** and **3c**). For the WM9 cell line, all the derivatives showed better inhibition than fangchinoline and vincristine. When comparing the “first stage” compounds to the “secondary stage” compounds, the halogenated “secondary stage” compounds (**4a** to **4f**) were weaker than their starting “first stage” compounds (**3a** and **3i**), while the nitrified compounds (**2a** and **4g**) were stronger than their starting compounds (**1a** and **3i**), with IC_50_ values of 3.04 µM and 1.07 µM, respectively. For the K562 cell line, the “secondary stage” compounds generally showed better inhibition than the “first stages” compounds. The halogenated “secondary stage” compounds showed better inhibition of cell growth than nitrified “secondary stage” compounds. For the MDA-MB-231 cell line, the inhibitory activities of those compounds were similar to their inhibitory activities against K562 cell line.

3 fangchinoline derivatives were also tested for their toxicities on normal human liver cell line HL7702. The IC_50_ values for compounds **3g** and **4g** against HL7702 cell growth were 17.26 µM and 5.00 µM, respectively, which were relatively 3 and 5 fold lower than their inhibition on cancer cell lines. Whereas, compound **2a** seemed more toxic against HL7702 cell (IC_50_ = 5.60 µM) than on cancer cell lines.

#### 2.2.2. The Annexin V-FITC/PI Flow Cytometry Assay

**Figure 2:**
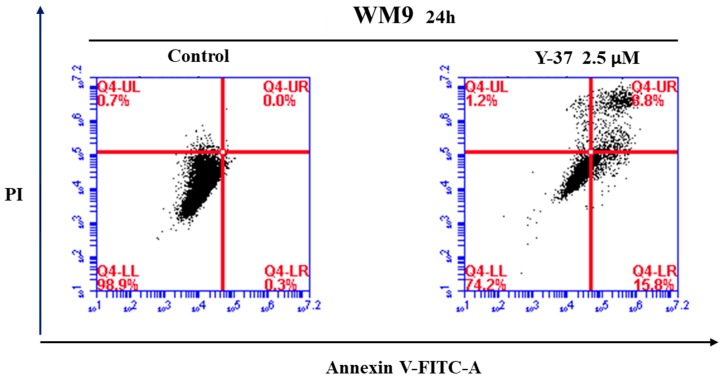
Compound **4g** induced apoptosis in WM9 cells. WM9 cells were stained with Annexin V-FITC and PI by incubating at room temperature for 15 min after treated cells with 2.5 µM **4g** for 24 h. Finally, apoptosis analysis was carried out by flow cytometry.

The Annexin V-FITC-A/PI apoptosis detection kit was induced to test the further role of compound **4g** in inhibiting WM9 cell death (Figure 2). Before undergoing the flow cytometry, the WM9 cells were treated with 0 and 2.5 µM compound **4g** for 24 h, and then Annexin V-FITC and PI were put into use. As shown in Figure 2, the WM9 cells which were treated with fangchinoline derivative **4g** showed 8.8% in the early apoptosis state and 15.8% in the late apoptosis state, which is significant higher than the control WM9 cells (0.0% and 0.3% in early and late apoptosis states, respectively). The flow cytometry result showed that compound **4g** could induce apoptosis in WM9 cell line.

#### 2.2.3. The Fangchinoline Derivative **4g** Affects the Protein Levels of Apoptosis Indicators in WM9 Cell Line

**Figure 3:**
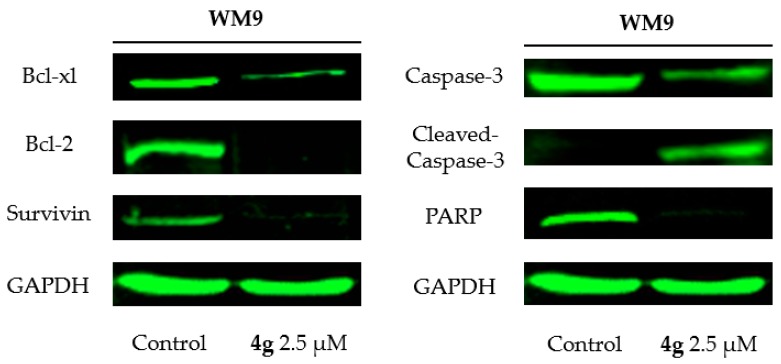
Fangchinoline derivative **4g** affects the protein levels of the apoptotic genes in WM9 cell line. Western blots using the indicated antibodies revealed that **4g** treatment activated the apoptosis pathway in WM9 cell line. WM9 cells were treated with 2.5 μM **4g** for 24 h.

Western blot analysis was induced to elucidate the effect of compound **4g** on protein levels to further expatiate how compound **4g** induces apoptosis in WM9 cells (Figure 3). The Bcl-xl, Bcl-2 and Survivin display repressor activities in cell death [29]. As shown in Figure 3, **4g** inhibited the Bcl-xl, Survivin and Bcl-2 expression, which is evidence of the activated mitochondrial pathway of cell apoptosis [30]. The activation of executioner from Pro-Caspase-3 to Cleaved-Caspase-3, which is also shown in Figure 3, means that apoptosis is being executed. The down-regulation of full-length PARP (the degradation of poly [ADP-ribose] polymerase), indirectly proved the up-regulation Cleaved-PARP, which could obliterate DNA repair and induce apoptosis in cells.

## 3. Materials and Methods

### 3.1. Method of Synthesis

The reagents and solvents were purchased commercially from Adamas and J&K chemical and were used without further purification unless otherwise noted. The solvents were purified according to the Guidelines in Purification of Laboratory Chemicals. Column chromatography was performed on silica gel (200–300 mesh, Qingdao, Shandong, China) using the indicated eluents. Thin-layer (0.25 mm, GF254) chromatography was carried out on silica gel plates (Qingdao, Shandong, China). NMR spectra were recorded on 400 MHz (Varian, Palo Alto, CA, USA) or spectrometers in appropriate solvents using TMS as internal standard or the solvent signals as secondary standards and the chemical shifts are shown in *δ* scales. Multiplicities of NMR signals were designated as s (singlet), d (doublet), t (triplet), br (broad), and m (multiplet, for unresolved lines). ^13^C-NMR spectra were recorded on 100 MHz or spectrometers. High-resolution mass spectra were obtained by using ESI-QTOF (Bruker, Billerica, MA, USA) mass spectrometry.

#### 3.1.1. General Procedure for the Synthesis of **1a** to **1c**

To a solution of fangchinoline (0.16 mmol, 1 eq.) in 5 mL dimethylformamide under protection of argon at ambient temperature, sodium hydride (0.2 mmol, 55%, 1.2 eq.) was added and stirred for 10 min. Then the halide (0.2 mmol, 1.2 eq.) was added dropwise and stirred for 8 to 12 h. The mixture was poured into ammonium chloride solution with ice water bath. The aqueous phase was extracted with 2 × 30 mL of dichloromethane. The combined organic phase was washed with brine and dried on magnesium sulfonate, followed by removal of the solvent by rotavapor. The crude mixture was purified over by column chromatography (silica, eluent: DCM:MeOH = 50:1) to afford the pure product.

*7-O-Benzyl-fangchinoline* (**1a**) white amorphous solid: yield 83%. C_44_H_46_N_2_O_6_ ESI-MS: *m*/*z* 698.86 [M + H]^+^; ^1^H-NMR (CDCl_3_, 400 MHz) *δ* (ppm): 7.34 (1H, dd, *J* = 2.0, 8.0 Hz), 7.22 (3H, m), 7.15 (1H, dd, *J* = 2.4, 8.0 Hz), 7.00 (2H, m), 6.88 (2H, m), 6.80 (1H, dd, *J* = 2.4, 8.0 Hz), 6.52 (1H, s), 6.51 (1H, s), 6.33 (1H, s), 6.31 (1H, d, *J* = 8.0 Hz), 5.86 (1H, s), 4.61 (1H, d, *J* = 10.4 Hz), 4.26 (1H, d, *J* = 10.4 Hz), 3.94 (3H, s), 3.76 (1H, m), 3.73 (3H, s), 3.51 (3H, m), 3.37 (3H, s), 3.31–2.55 (8H, m), 2.53 (2H, m), 2.50 (3H, s), 2.35 (3H, s); ^13^C-NMR (CDCl_3_, 100 MHz) *δ* (ppm): 154.0, 151.6, 149.5, 148.9, 148.8, 147.2, 144.2, 137.6, 136.7, 134.7, 132.7, 130.3, 128.4, 128.4, 128.1, 128.1, 128.1, 128.1, 128.1, 127.7, 127.7, 127.7, 123.0, 122.1, 122.1, 120.5, 116.2, 112.9, 111.6, 106.1, 74.5, 64.2, 61.7, 56.2, 56.0, 55.9, 45.5, 42.5, 42.3, 42.0, 40.3, 37.2, 24.5, 22.8.

*7-O-(3-Methylbut-1-ene)-fangchinoline* (**1b**) off-yellow amorphous solid: yield 57%. C_42_H_48_NO_6_ ESI-MS: *m*/*z* 676.85 [M + H]^+^; ^1^H-NMR (CDCl_3_, 400 MHz) *δ* (ppm): 7.37 (1H, dd, *J* = 2.0, 8.0 Hz), 7.15 (1H, dd, *J* = 2.4, 8.0 Hz), 6.92 (1H, d, *J* = 8.0 Hz, H-14), 6.88 (1H, s), 6.86 (1H, s), 6.83 (1H, dd, *J* = 2.4, 8.4 Hz), 6.54 (1H, d, *J* = 1.6 Hz), 6.51 (1H, s), 6.33 (1H, d, *J* = 2.4 Hz), 6.31 (1H, s), 5.96 (1H, s), 4.83 (1H, dd, *J* = 6.4, 7.6 Hz), 4.00 (1H, m), 3.94 (3H, s), 3.84 (1H, m), 3.75 (3H, s), 3.55 (2H, m), 3.37 (3H, s), 3.33 (1H, m), 2.97-2.70 (8H, m), 2.64 (3H, s), 2.52 (2H, m), 2.35 (3H, s), 1.60 (3H, s, –CH_3_–), 1.52 (3H, s); ^13^C-NMR (CDCl_3_, 100 MHz) *δ* (ppm): 153.8, 151.7, 149.4, 148.8, 148.7, 147.1, 143.9, 136.8, 136.6, 135.2, 134.8, 132.8, 130.3, 128.1, 128.1, 127.3, 122.8, 122.1, 122.0, 120.6, 120.3, 116.1, 112.7, 111.5, 105.6, 69.3, 64.5, 61.6, 56.2, 55.9, 55.8, 45.7, 44.3, 42.7, 42.4, 42.0, 41.0, 29.8, 25.8, 24.4, 22.8, 22.2.

*7-O-(Prop-1-ene)-fangchinoline* (**1c**) off-yellow amorphous solid: yield 71%. C_40_H_44_N_2_O_6_ ESI-MS: *m*/*z* 648.80 [M + H]^+^; ^1^H-NMR (CDCl_3_, 400 MHz) *δ* (ppm): 7.37 (1H, d, *J* = 8.4 Hz, H-14′), 7.15 (1H, dd, *J* = 2.4, 8.4 Hz), 6.87 (2H, s, H-13), 6.82 (1H, dd, *J* = 2.4, 8.4 Hz), 6.54 (1H, s), 6.51 (1H, s), 6.33 (1H, dd, *J* = 2.4, 8.4 Hz), 6.31 (1H, s), 5.96 (1H, s), 5.45 (1H, m), 4.95 (2H, m), 4.05 (1H, dd, *J* = 5.2, 11.6 Hz), 3.93 (3H, s), 3.86 (1H, dd, *J* = 6.0, 11.2 Hz), 3.78 (2H, m), 3.74 (3H, s), 3.53 (2H, m), 3.37 (3H, s), 3.01–2.68 (8H, m), 2.60 (3H, s), 2.48 (2H, m), 2.33 (3H, s); ^13^C-NMR (CDCl_3_, 100 MHz) *δ* (ppm): 153.6, 151.4, 149.3, 148.5, 148.4, 146.9, 143.7, 136.3, 135.0, 134.6, 133.9, 132.6, 130.1, 128.2, 128.0, 127.4, 122.8, 122.7, 121.9, 121.8, 120.3, 116.8, 115.9, 112.6, 111.3, 105.6, 73.4, 64.2, 61.3, 56.0, 55.7, 55.6, 45.4, 44.0, 42.5, 42.2, 41.8, 40.7, 24.1, 22.0.

#### 3.1.2. General Procedure for the Synthesis of **3a** to **3i**

To a solution of fangchinoline (0.16 mmol, 1 eq.) in 5 mL dichloromethane with the protection of argon at ambient temperature, acyl halide (0.18 mmol, 1.1 eq.) and DMAP (0.04 mmol, 0.25 eq.) were added and stirred for 1.5 h. The reaction was poured into 10 mL saturated sodium bicarbonate solution and exacted with 2 × 30 mL of dichloromethane. The combined organic phase was washed with brine and dried by magnesium sulfonate, followed by removal of the solvent by rotavapor. The residue was purified by column chromatography (silica, eluent: DCM:MeOH = 50:1) to afford the pure product.

*7-O-Acetyl-fangchinoline* (**3a**) off-white amorphous solid: yield 96%. C_39_H_42_N_2_O_7_ ESI-MS: *m*/*z* 650.77 [M + H]^+^; ^1^H-NMR (CDCl_3_, 400 MHz) *δ* (ppm): 7.33 (1H, dd, *J* = 2.0, 8.4 Hz), 7.13 (1H, dd, *J* = 2.8, 8.4 Hz), 6.86 (1H, s), 6.85 (1H, s), 6.80 (1H, dd, *J* = 2.4, 8.4 Hz), 6.57 (1H, s), 6.52 (1H, s), 6.32 (1H, dd, *J* = 2.0, 8.4 Hz), 6.28 (1H, s), 6.05 (1H, s), 3.92 (3H, s), 3.88 (1H, dd, *J* = 5.6, 11.2 Hz), 3.76 (3H, s), 3.74–3.36 (3H, m), 3.35 (3H, s), 3.23 (1H, dd, *J* = 6.0, 12.4 Hz), 3.00–2.69 (7H, m), 2.61 (3H, s), 2.55–2.34 (2H, m), 2.28 (3H, s), 2.32 (3H, s); ^13^C-NMR (CDCl_3_, 100 MHz) *δ* (ppm): 163.9, 154.3, 151.9, 148.7, 147.6, 147.2, 146.5, 141.3, 140.1, 135.7, 133.5, 132.5, 130.8, 129.1, 127.8, 122.1, 121.9, 121.8, 121.5, 120.1, 115.8, 111.6, 104.3, 63.6, 61.9, 56.2, 55.4, 50.8, 45.6, 44.1, 42.6, 42.1, 41.8, 39.7, 29.7, 25.5, 23.3.

*7-O-Tosyl-fangchinoline* (**3b**) yellow amorphous solid: yield 90%. C_44_H_46_N_2_O_8_S ESI-MS: *m*/*z* 762.29 [M + H]^+^; ^1^H-NMR (CDCl_3_, 400 MHz) *δ* (ppm): 7.75 (2H, s), 7.50 (2H, s), 7.47 (2H, t, *J* = 15.2 Hz), 7.35 (2H, d, *J* = 6.6 Hz), 7.15 (2H, d, *J* = 6.4 Hz), 7.10 (1H, s), 6.92 (1H, s), 6.83 (3H, m), 6.52 (1H, s), 4.57 (2H, m), 3.92 (3H, s), 3.90 (6H, s), 3.81(3H, s), 3.00 (4H, m), 3.48 (3H, s), 3.41–2.36 (9H, m), 2.17 (3H, s), 1.89 (2H, m), 1.26 (3H, s); ^13^C-NMR (CDCl_3_, 100 MHz) *δ* (ppm): 155.0, 149.9, 148.5, 148.0, 147.1, 144.2, 144.0, 138.8, 134.9, 132.7, 130.4, 130.0, 130.0, 128.9, 128.9, 128.3, 128.3, 128.3, 128.0, 128.0, 122.8, 122.8, 122.3, 122.2, 121.8, 120.7, 116.0, 112.8, 111.5, 105.7, 64.0, 61.4, 56.2, 56.2, 56.1, 45.6, 44.0, 42.4, 41.9, 41.6, 41.3, 29.8, 24.6, 22.8.

*7-O-(o-Nirtobenzessulfonyl)-fangchinoline* (**3c**) yellow amorphous solid: yield 90%. C_44_H_46_N_3_O_10_S ESI-MS: *m*/*z* 793.89 [M + H]^+^; ^1^H-NMR (CDCl_3_, 400 MHz) *δ* (ppm): 8.50 (1H, dd, *J* = 2.0, 8.0 Hz), 8.01–8.05 (3H, m), 7.35 (2H, dd, *J* = 2.6, 8.6 Hz), 7.15 (2H, dd, *J* = 2.4, 8.4 Hz), 7.10 (1H, s), 6.92 (1H, s), 6.83 (3H, m), 6.52 (1H, s), 4.57 (2H, m), 3.92 (3H, s), 3.90 (6H, s), 3.81(3H, s), 3.00 (4H, m), 3.48 (3H, s), 3.41–2.36 (9H, m), 2.17 (3H, s), 1.89 (2H, m), 1.26 (3H, s); ^13^C-NMR (CDCl_3_, 100 MHz) *δ* (ppm): 155.0, 149.9, 148.5, 148.0, 148.0, 147.1, 144.2, 144.0, 138.8, 136.3, 134.9, 133.4, 130.4, 128.9, 128.9, 128.3, 128.3, 128.3, 128.0, 125.2, 122.8, 122.8, 122.3, 122.2, 121.8, 120.7, 116.0, 112.8, 111.5, 105.7, 64.0, 61.4, 56.2, 56.2, 56.1, 45.6, 44.0, 42.4, 41.9, 41.6, 41.3, 29.8, 24.6, 22.8.

*7-O-(p-Nitro-benzoyl)-fangchinoline* (**3d**) yellow amorphous solid: 90%. C_44_H_43_N_3_O_9_ ESI-MS: *m*/*z* 757.84 [M + H]^+^; ^1^H-NMR (CDCl3, 400 MHz) *δ* (ppm): 8.30–8.38 (4H, m), 7.35 (2H, dd, *J* = 2.2, 8.2 Hz), 7.15 (2H, dd, *J* = 2.4, 8.4 Hz), 7.10 (1H, s), 6.90 (1H, s), 6.83 (3H, m), 6.52 (1H, s), 4.57 (2H, m), 3.92 (3H, s), 3.90 (6H, s), 3.81(3H, s), 3.00 (4H, m), 3.48 (3H, s), 2.36–3.41 (9H, m), 2.17 (3H, s), 1.89 (2H, m), 1.26 (3H, s); ^13^C-NMR (CDCl_3_, 100 MHz) *δ* 165.2, 154.2, 153.1, 149.9, 149.6, 148.3, 148.1, 147.2, 144.9, 133.6, 132.7, 132.7, 132.3, 131.3, 131.3, 128.5, 128.5, 128.2 127.8, 126.1, 123.8, 123.8, 123.0, 123.0, 121.6, 121.6, 119.5, 117.8, 110.2, 110.1, 105.7, 64.0, 61.4, 56.2, 56.2, 56.1, 45.6, 44.0, 42.4, 41.9, 41.6, 41.3, 29.8, 22.8.

*7-O-(p-Methyl-benzoyl)-fangchinoline* (**3e**) brown amorphous solid: yield 95%. C_45_H_46_N_2_O_7_ ESI-MS: *m*/*z* 726.86 [M + H]^+^; ^1^H-NMR (CDCl_3_, 400 MHz) *δ* (ppm): 8.03 (1H, dd, *J* = 2.4, 8.4 Hz), 7.42 (2H, dd, *J* = 2.0, 8.0 Hz), 7.35 (2H, dd, *J* = 2.0, 8.0 Hz), 7.15 (2H, dd, *J* = 2.0, 8.0 Hz), 7.10 (1H, s), 6.90 (1H, s), 6.83 (3H, m), 6.52 (1H, s), 4.57 (2H, m), 3.92 (3H, s), 3.90 (6H, s), 3.81(3H, s), 3.00 (4H, m), 3.48 (3H, s), 3.41–2.36 (9H, m), 2.41 (3H, s), 2.17 (3H, s), 1.89 (2H, m), 1.26 (3H, s); ^13^C-NMR (CDCl_3_,100 MHz) *δ* (ppm): 165.2, 154.2, 153.1, 149.9, 149.6, 148.3, 148.1, 147.2, 144.9, 133.6, 132.7, 132.7, 132.3, 130.2, 130.2, 128.8, 128.8, 128.2 127.8, 126.1, 123.8, 123.8, 123.0, 123.0, 121.6, 121.6, 119.5, 117.8, 110.2, 110.1, 105.7, 64.0, 61.4, 56.2, 56.2, 56.1, 45.6, 44.0, 42.4, 41.9, 41.6, 41.3, 29.8, 22.8.

*7-O-(p-Methoxy-benzoyl)-fangchinoline* (**3f**) yellow amorphous solid: yield 91%. C_45_H_46_N_2_O_8_ ESI-MS: *m*/*z* 742.87 [M + H]^+^; ^1^H-NMR (CDCl_3_, 400 MHz) *δ* (ppm): 8.13 (2H, dd, *J* = 2.0, 8.0 Hz), 7.42 (2H, dd, *J* = 2.2, 8.2 Hz), 7.35 (2H, dd, *J* = 2.0, 8.0 Hz), 7.15 (2H, dd, *J* = 2.0, 8.0 Hz), 7.10 (1H, s), 6.90 (1H, s), 6.83 (3H, m), 6.52 (1H, s), 4.57 (2H, m), 3.92 (3H, s), 3.90 (6H, s), 3.83 (3H, s), 3.81(3H, s), 3.00 (4H, m), 3.48 (3H, s), 3.41–2.36 (9H, m), 2.41 (3H, s), 2.17 (3H, s), 1.89 (2H, m), 1.26 (3H, s); ^13^C-NMR (CDCl_3_, 100 MHz) *δ* (ppm): 163.8, 153.7, 149.5, 147.0, 142.8, 134.9, 134.8, 133.1, 132.7, 132.5, 131.2, 131.2, 130.4, 128.3, 128.3, 128.3, 128.0, 128.0, 122.8, 122.8, 122.3, 122.2, 121.8, 120.7, 116.0, 114.2, 114.2, 112.8, 111.5, 105.7, 64.0, 61.4, 56.2, 56.2, 56.1, 45.6, 44.0, 42.4, 41.9, 41.6, 41.3, 29.8, 24.6, 22.8.

*7-O-(Furan-m-formyl)-fangchinoline* (**3g**) yellow amorphous solid: yield 90%. C_44_H_44_N_2_O_7_ ESI-MS: *m*/*z* 702.80 [M + H]^+^; ^1^H-NMR (CDCl_3_, 400 MHz) *δ* (ppm): 7.97 (1H, m), 7.63 (1H, m), 7.35 (2H, dd, *J* = 2.0, 8.0 Hz), 7.15 (2H, dd, *J* = 2.0, 8.0 Hz), 7.10 (1H, s), 6.90 (1H, s), 6.83 (3H, m), 6.52 (1H, s), 4.57 (2H, m), 3.92 (3H, s), 3.90 (6H, s), 3.83 (3H, s), 3.81(3H, s), 3.00 (4H, m), 3.48 (3H, s), 3.41–2.36 (9H, m), 2.41 (3H, s), 2.17 (3H, s), 1.89 (2H, m), 1.26 (3H, s); ^13^C-NMR (CDCl_3_, 100 MHz) *δ* (ppm): 177.5, 153.7, 149.5, 147.0, 145.6, 142.8, 134.9, 134.8, 133.1, 132.7, 132.5, 130.4, 130.0, 130.0, 128.3, 128.3, 128.3, 128.0, 128.0, 122.8, 122.8, 122.3, 122.2, 121.8, 120.7, 118.6, 116.0, 112.8, 112.0, 111.5, 105.7, 64.0, 61.4, 56.2, 56.2, 56.1, 45.6, 44.0, 42.4, 41.9, 41.6, 41.3, 29.8, 24.6, 22.8.

*7-O-Propionyl-fangchinoline* (**3h**) light yellow amorphous solid: yield 88%. C_40_H_44_N_2_O_7_, ESI-MS: *m*/*z* 664.80 [M + H]^+^; ^1^H-NMR (CDCl_3_, 400 MHz) *δ* (ppm): 7.33 (1H, dd, *J* = 2.0, 8.0 Hz), 7.13 (1H, dd, *J* = 2.0, 8.0 Hz), 6.86 (1H, s), 6.80 (1H, dd, *J* = 2.0, 8.0 Hz), 6.52 (1H, s), 6.48 (1H, s), 6.43 (1H, s), 6.24 (1H, d, *J* = 8.0 Hz), 5.91 (1H, s), 5.68 (1H, s), 3.93 (3H, s), 3.72 (1H, m), 3.61 (1H, dd, *J* = 5.6, 11.2 Hz), 3.00 (3H, s), 2.85 (1H, m), 2.73 (3H, m), 2.64 (3H, m), 2.43 (3H, s); ^13^C-NMR (CDCl_3_, 100 MHz) *δ* (ppm): 172.6, 153.7, 149.5, 147.0, 142.8, 134.9, 134.8, 133.1, 132.7, 132.5, 130.4, 130.0, 130.0, 128.3, 128.3, 128.3, 128.0, 128.0, 122.8, 122.8, 122.3, 122.2, 121.8, 120.7, 116.0, 112.8, 111.5, 105.7, 64.0, 61.4, 56.2, 56.2, 56.1, 45.6, 44.0, 42.4, 41.9, 41.6, 41.3, 29.8, 27.8, 24.6, 22.8, 11.5.

*7-O-Benzoyl-fangchinoline* (**3i**) light yellow amorphous solid: yield 97%. C_44_H_44_N_2_O_7_ ESI-MS: *m*/*z* 712.84 [M + H]^+^; ^1^H-NMR (CDCl_3_, 400 MHz) *δ* (ppm): 8.16 (2H, dd, *J* = 2.0, 8.0 Hz), 7.78 (1H, s), 7.47 (2H, t, *J* = 15.2 Hz), 7.34 (2H, m), 7.10 (1H, s), 6.87 (1H, s), 6.77 (1H, m), 6.60 (1H, s), 6.44 (1H, s), 6.39 (1H, s), 6.24 (1H, m), 5.57 (1H, s), 3.92 (3H, s), 3.69 (3H, s), 3.55 (3H, m), 3.48 (3H, s), 3.41–2.36 (9H, m), 2.17 (3H, s), 1.89 (2H, m), 1.26 (3H, s); ^13^C-NMR (CDCl_3_, 100 MHz) *δ* (ppm): 163.8, 153.7, 149.5, 147.0, 142.8, 134.9, 134.8, 133.1, 132.7, 132.5, 130.4, 130.0, 130.0, 128.9, 128.9, 128.3, 128.3, 128.3, 128.0, 128.0, 122.8, 122.8, 122.3, 122.2, 121.8, 120.7, 116.0, 112.8, 111.5, 105.7, 64.0, 61.4, 56.2, 56.2, 56.1, 45.6, 44.0, 42.4, 41.9, 41.6, 41.3, 29.8, 24.6, 22.8.

#### 3.1.3. General Procedure for the Synthesis of **4a** to **4h**

*7-O-Benzoyl-fangchinoline* (**3i**, 0.16 mmol, 1 eq.) or *7-O-Acetyl-fangchinoline* (**3a**, 0.16 mmol, 1 eq.) was added to 5 mL of trifluoracetic acid at 0 °C, and *N*-bromosuccinimide (0.18 mmol, 1.1 eq.) was added all at once and stirred for 10 min. Then, the reaction mixture was warmed up to room temperature and stirred for another 3 h. The mixture was then poured into ice-water and aqueous sodium bicarbonate was added dropwise until pH was about 8. The aqueous phase was exacted with 2 × 30 mL of dichloromethane. The combined organic phase was washed with brine and dried by magnesium sulfonate, followed by removal of the solvent by rotavapor. The crude mixture was purified by column chromatography (silica, eluent: DCM:MeOH = 30:1) to afford compound **4c**.

For compound **4f**, 1.1 eq. of *N*-chlorosuccinimide was added, while the other procedures were the same.

For compounds **4a**, **4b**, **4d** and **4e**, 2.2 eq. of *N*-bromosuccinimide or *N*-chlorosuccinimide was added, while the other procedures were the same.

*7-O-Benzoyl-5,14-dibromo-fangchinoline* (**4a**) brown amorphous solid: yield 63%. C_44_H_42_Br_2_N_2_O_7_ ESI-MS: *m*/*z* 870.64 [M + H]^+^; ^1^H-NMR (CDCl_3_, 400 MHz) *δ* (ppm): 8.16 (2H, dd, *J* = 2.0, 8.0 Hz), 7.78 (1H, s), 7.47 (2H, t, *J* = 15.2 Hz), 7.34 (2H, m), 7.10 (1H, s), 6.60 (1H, s), 6.44 (1H, s), 6.39 (1H, s), 6.24 (1H, m), 5.57 (1H, s), 3.92 (3H, s), 3.69 (3H, s), 3.55 (3H, m), 3.48 (3H, s), 3.41–2.36 (9H, m), 2.17 (3H, s), 1.89 (2H, m), 1.26 (3H, s); ^13^C-NMR (CDCl_3_, 100 MHz) *δ* (ppm): 163.8, 153.7, 149.5, 147.0, 142.8, 134.9, 134.8, 133.1, 132.7, 132.5, 130.4, 130.0, 130.0, 128.9, 128.9, 128.3, 128.3, 128.3, 128.0, 128.0, 122.8, 122.3, 122.2, 121.8, 120.7, 116.0, 115.7, 112.8, 112.5, 111.5, 64.0, 61.4, 56.2, 56.2, 56.1, 45.6, 44.0, 42.4, 41.9, 41.6, 41.3, 29.8, 24.6, 22.8.

*7-O-Benzoyl-5,14-dichloro-fangchinoline* (**4b**) dark yellow amorphous solid: yield 79%. C_44_H_42_Cl_2_N_2_O_7_ ESI-MS: *m*/*z* 781.73 [M + H]^+^; ^1^H-NMR (CDCl_3_, 400 MHz) *δ* (ppm): 8.16 (2H, dd, *J* = 2.0, 8.0 Hz), 7.78 (1H, s), 7.47 (2H, t, *J* = 15.2 Hz), 7.34 (2H, m), 7.10 (1H, s), 6.60 (1H, s), 6.44 (1H, s), 6.39 (1H, s), 6.24 (1H, m), 5.57 (1H, s), 3.92 (3H, s), 3.69 (3H, s), 3.55 (3H, m), 3.48 (3H, s), 3.41–2.36 (9H, m), 2.17 (3H, s), 1.89 (2H, m), 1.26 (3H, s); ^13^C-NMR (CDCl_3_, 100 MHz) *δ* (ppm): 163.8, 153.7, 149.5, 147.0, 142.8, 134.9, 134.8, 133.1, 132.7, 132.5, 130.4, 130.0, 130.0, 128.9, 128.9, 128.3, 128.3, 128.3, 128.0, 128.0, 122.8, 122.3, 122.2, 121.8, 120.7, 116.0, 114.7, 114.1, 112.8, 111.5, 64.0, 61.4, 56.2, 56.2, 56.1, 45.6, 44.0, 42.4, 41.9, 41.6, 41.3, 29.8, 24.6, 22.8.

*7-O-Acetyl-5-bromo-fangchinoline* (**4c**) light yellow amorphous solid: yield 75%. C_39_H_41_BrN_2_O_7_ ESI-MS: *m*/*z* 729.67 [M + H]^+^; ^1^H-NMR (CDCl_3_, 400 MHz) *δ* (ppm): 7.33 (1H, dd, *J* = 2.0, 8.4 Hz), 7.13 (1H, dd, *J* = 2.8, 8.4 Hz), 6.86 (1H, s), 6.85 (1H, s), 6.80 (1H, dd, *J* = 2.4, 8.4 Hz), 6.52 (1H, s), 6.32 (1H, dd, *J* = 2.0, 8.4 Hz), 6.28 (1H, s), 6.05 (1H, s), 3.92 (3H, s), 3.88 (1H, dd, *J* = 5.6, 11.2 Hz), 3.76 (3H, s), 3.74–3.36 (3H, m), 3.35 (3H, s), 3.23 (1H, dd, *J* = 6.0, 12.4 Hz), 3.00–2.69 (7H, m), 2.61 (3H, s), 2.55–2.34 (2H, m), 2.28 (3H, s), 2.32 (3H, s); ^13^C-NMR (CDCl_3_, 100 MHz) *δ* (ppm): 163.9, 154.3, 151.9, 148.7, 147.6, 147.2, 146.5, 141.3, 140.1, 135.7, 133.5, 132.5, 130.8, 129.1, 127.8, 122.1, 121.9, 121.8, 121.5, 120.1, 115.8, 112.5, 111.6, 63.6, 61.9, 56.2, 55.4, 50.8, 45.6, 44.1, 42.6, 42.1, 41.8, 39.7, 29.7, 25.5, 23.3.

*7-O-Acetyl-5,14-dibromo-fangchinoline* (**4d**) yellow amorphous solid: yield 50%. C_39_H_40_Br_2_N_2_O_7_ ESI-MS: *m*/*z* 808.56 [M + H]^+^; ^1^H-NMR (CDCl_3_, 400 MHz) *δ* (ppm): 7.33 (1H, dd, *J* = 2.0, 8.4 Hz), 7.13 (1H, dd, *J* = 2.8, 8.4 Hz), 6.86 (1H, s), 6.80 (1H, dd, *J* = 2.4, 8.4 Hz), 6.52 (1H, s), 6.32 (1H, dd, *J* = 2.0, 8.4 Hz), 6.28 (1H, s), 6.05 (1H, s), 3.92 (3H, s), 3.88 (1H, dd, *J* = 5.6, 11.2 Hz), 3.76 (3H, s), 3.74–3.36 (3H, m), 3.35 (3H, s), 3.23 (1H, dd, *J* = 6.0, 12.4 Hz), 3.00–2.69 (7H, m), 2.61 (3H, s), 2.55–2.34 (2H, m), 2.28 (3H, s), 2.32 (3H, s); ^13^C-NMR (CDCl_3_, 100 MHz) *δ* (ppm): 163.9, 154.3, 151.9, 148.7, 147.6, 147.2, 146.5, 141.3, 140.1, 135.7, 133.5, 132.5, 130.8, 129.1, 127.8, 122.1, 121.8, 121.5, 120.1, 115.8, 115.0, 112.5, 111.6, 63.6, 61.9, 56.2, 55.4, 50.8, 45.6, 44.1, 42.6, 42.1, 41.8, 39.7, 29.7, 25.5, 23.3.

*7-O-Acetyl-5,14-dichloro-fangchinoline* (**4e**) yellow amorphous solid: yield 43%. C_39_H_40_Cl_2_N_2_O_7_ ESI-MS: *m*/*z* 719.66 [M + H]^+^; ^1^H-NMR (CDCl_3_, 400 MHz) *δ* (ppm): 7.33 (1H, dd, *J* = 2.0, 8.4 Hz), 7.13 (1H, dd, *J* = 2.8, 8.4 Hz), 6.85 (1H, s), 6.80 (1H, dd, *J* = 2.4, 8.4 Hz), 6.52 (1H, s), 6.32 (1H, dd, *J* = 2.0, 8.4 Hz), 6.28 (1H, s), 6.05 (1H, s), 3.92 (3H, s), 3.88 (1H, dd, *J* = 5.6, 11.2 Hz), 3.76 (3H, s), 3.74–3.36 (3H, m), 3.35 (3H, s), 3.23 (1H, dd, *J* = 6.0, 12.4 Hz), 3.00–2.69 (7H, m), 2.61 (3H, s), 2.55–2.34 (2H, m), 2.28 (3H, s), 2.32 (3H, s); ^13^C-NMR (CDCl_3_, 100 MHz) *δ* (ppm): 163.9, 154.3, 151.9, 148.7, 147.6, 147.2, 146.5, 141.3, 140.1, 135.7, 133.5, 132.5, 130.8, 129.1, 127.7, 122.1, 121.8, 121.5, 120.1, 115.8, 114.1, 112.5, 111.6, 63.6, 61.9, 56.2, 55.4, 50.8, 45.6, 44.1, 42.6, 42.1, 41.8, 39.7, 29.7, 25.5, 23.3.

*7-O-Acetyl-5-chloro-fangchinoline* (**4f**) yellow amorphous solid: yield 69%. C_39_H_41_BrN_2_O_7_ ESI-MS: *m*/*z* 729.67 [M + H]^+^; ^1^H-NMR (CDCl_3_, 400 MHz) *δ* (ppm): 7.33 (1H, dd, *J* = 2.0, 8.4 Hz), 7.13 (1H, dd, *J* = 2.8, 8.4 Hz), 6.86 (1H, s), 6.85 (1H, s), 6.80 (1H, dd, *J* = 2.4, 8.4 Hz), 6.52 (1H, s), 6.32 (1H, dd, *J* = 2.0, 8.4 Hz), 6.28 (1H, s), 6.05 (1H, s), 3.92 (3H, s), 3.88 (1H, dd, *J* = 5.6, 11.2 Hz), 3.76 (3H, s), 3.74–3.36 (3H, m), 3.35 (3H, s), 3.23 (1H, dd, *J* = 6.0, 12.4 Hz), 3.00–2.69 (7H, m), 2.61 (3H, s), 2.55–2.34 (2H, m), 2.28 (3H, s), 2.32 (3H, s); ^13^C-NMR (CDCl_3_, 100 MHz) *δ* (ppm): 163.9, 154.3, 151.9, 148.7, 147.6, 147.2, 146.5, 141.3, 140.1, 135.7, 133.5, 132.5, 130.8, 129.1, 127.8, 122.1, 121.9, 121.8, 121.5, 120.1, 115.8, 112.5, 111.6, 63.6, 61.9, 56.2, 55.4, 50.8, 45.6, 44.1, 42.6, 42.1, 41.8, 39.7, 29.7, 25.5, 23.3.

#### 3.1.4. General Procedure for the Synthesis of Compounds **2a** and **4g**

Under the protection of argon, concentrated nitric acid (0.6 mL) was carefully dropped into the acetic anhydride (1.5 mL) in a pre-dried 10 mL round flask and stirred for 5 min at −10 °C to obtain the mixed acid. *7-Benzyl-fangchinoline* (**1a**, 0.80 mmol, 1 eq.) or *7-O-Benzoyl-fangchinoline* (**3i**, 0.80 mmol, 1eq.) dissolved in 3 mL dichloromethane was added into the mixed acid dropwise. The reaction was allowed to stir at room temperature for 70 min before 10 mL of water was added. The aqueous phase was exacted with 2 × 10 mL of dichloromethane. The combined organic phase was washed with brine and dried over magnesium sulfonate. The solvents were removed by rotavapor. The crude mixture was purified by column chromatography (silica, eluent: DCM:MeOH = 40:1) to afford the pure product.

*7-Benzyl-14-nitro-fangchinoline* (**2a**) yellow amorphous solid: yield 68%. C_44_H_45_N_3_O_8_ ESI-MS: *m*/*z* 743.86 [M + H]^+^; ^1^H-NMR (CDCl_3_, 400 MHz) *δ* (ppm): 7.42 (1H, s), 7.37 (1H, dd, *J* = 2.0, 8.0 Hz), 7.22 (1H, m), 7.21 (2H, d, *J* = 2.0 Hz), 7.13 (1H, dd, *J* = 2.4, 8.0 Hz), 6.99 (2H, m), 6.75 (1H, dd, *J* = 2.4, 8.0 Hz), 6.52 (1H, s), 6.51 (1H, s), 6.33 (1H, s), 6.29 (1H, dd, *J* = 2.0, 8.4 Hz), 5.85 (1H, s), 4.58 (1H, d, *J* = 10.8 Hz), 4.28 (1H, d, *J* = 10.8 Hz), 3.99 (3H, s), 3.81 (2H, m), 3.72 (3H, s), 3.59 (2H, m), 3.38 (3H, s), 3.32–2.69 (8H, m), 2.54 (1H, d, *J* = 13.6 Hz), 2.49 (3H, s), 2.35 (1H, dd, *J* = 4.0, 16.8 Hz), 2.22 (3H, s); ^13^C-NMR (CDCl_3_, 100 MHz) *δ* (ppm): 154.0, 153.3, 151.1, 148.6, 147.8, 144.2, 142.2, 138.6, 136.7, 132.5, 129.0, 129.0, 128.5, 128.5, 128.0, 127.9, 127.9, 127.8, 127.8, 127.7, 127.7, 127.7, 123.0, 122.1, 122.1, 120.5, 116.2, 112.9, 111.6, 106.1, 74.5, 64.2, 61.7, 56.2, 56.0, 55.9, 45.5, 42.5, 42.3, 42.0, 40.3, 37.2, 24.5, 22.8.

*7-O-Benzoyl-14-nitro-fangchinoline* (**4g**) light brown amorphous solid: yield 85%. C_44_H_42_Br_2_N_2_O_7_ ESI-MS: *m*/*z* 757.84 [M + H]^+^; ^1^H-NMR (CDCl_3_, 400 MHz) *δ* (ppm): 8.16 (2H, dd, *J* = 2.0, 8.0Hz), 7.78 (1H, s), 7.47 (2H, t, *J* = 15.2 Hz), 7.34 (2H, m), 6.66 (1H, s), 6.60 (1H, s), 6.44 (1H, s), 6.39 (1H, s), 6.24 (1H, m), 5.57 (1H, s), 3.92 (3H, s), 3.69 (3H, s), 3.55 (3H, m), 3.48 (3H, s), 3.41–2.36 (9H, m), 2.17 (3H, s), 1.89 (2H, m), 1.26 (3H, s); ^13^C-NMR (CDCl_3_, 100 MHz) *δ* (ppm): 163.6, 154.1, 152.9, 151.0, 149.4, 149.1, 148.8, 144.1, 142.7, 133.5, 132.4, 130.0, 130.0, 128.6, 128.6, 128.3, 128.3, 128.3, 128.0, 128.0, 122.8, 122.8, 122.3, 122.2, 121.8, 120.7, 116.0, 112.8, 111.5, 105.7, 64.0, 61.4, 56.2, 56.2, 56.1, 45.6, 44.0, 42.4, 41.9, 41.6, 41.3, 29.8, 24.6, 22.8.

### 3.2. Cell Lines and Cell Culture

Human erythroleukemic cell lines HEL and K562, human breast cell lines MDA-MB-231, human prostate cell lines PC3 and human melanoma cell lines WM9 were cultured in RPMI (HEL and K562) or DMEM (MDA-MB-231, PC3, WM9) medium (high glucose) supplemented with 5% FBS (HyClone, GE Healthcare, Parramatta, Australia) and 1% ampicillin/streptomycin.

### 3.3. In Vitro Drug Studies

Cells were cultured in 96-well plates at densities of 1 × 10^5^/well. The plates were pre-incubated at 37 °C for 24 h to allow adaptation of cells prior to the addition of the test compounds. Then, compounds were added to cells in triplicate in several doses, and incubated for an additional 2 days. The 50% inhibitory concentrations (IC_50_) were measured for fangchinoline derivatives using MTT (diphenyltetrazolium bromide) assay.

### 3.4. Flow Cytometry

For apoptosis analysis, WM9 cells were incubated with compounds or DMSO as a vehicle control for 24 h. Cells were then washed with cold PBS, stained with Annexin V-FITC and Propidium Iodide (PI) using apoptosis detection Kit (BD Biosciences, Bergen, NJ, USA).

### 3.5. Western Blotting

The total protein was collected from WM9 cells using RIPA lysis buffer, and separated on 10% SDS Page, as described [31]. The protein was then transferred to polyvinylidene fluoride (PVDF, 0.2 µm) membrane, and blocked by 5% BSA solution for 2 h at room temperature. The membrane was then incubated with specific primary antibodies overnight at 4 °C, followed by incubation with corresponding HRP-conjugated secondary antibodies for 2 h at room temperature. Expression of particular proteins was measured by applying ECL select substrate (Li-Cor, Lincoln, NE, USA). Polyclonal rabbit antibody for Bcl-2 was obtained from Abcam (Abcam, Cambridge, UK); Bcl-xl, Survivin, Casepase-3 and PARP antibodies from Cell Signalling Technology (CST, Danvers, MA, USA) and GAPDH from Goodhere Biotechnology (Hangzhou, Zhejiang, China).

## 4. Conclusions

In conclusion, 12 “first stage” and 8 “secondary stage” fangchinoline derivatives were designed and synthesized. The evaluation of their anti-cancer activity against HEL, PC3, WM9, K562, MDA-MB-231 cell lines were performed, and the results showed that the inhibitory activities of fangchinoline derivatives were significantly higher than those of the original compound fangchinoline and the positive control vincristine. Among them, the “secondary stage” derivative **4g** exhibited the best inhibition activity on the WM9 cell line, with IC_50_ of values 1.07 µM, which is 2.5 times more active than the original “first stage” derivative **3i**, 12.2 times more active than fangchinoline, and 9.8 times more active than vincristine. The SARs analysis showed that all of the fangchinoline derivatives were able to improve anti-cancer activities. Moreover, most of the “secondary stage” compounds showed improved anti-cancer activities in comparison to the “first stage” derivatives in PC3 cells, WM9 cells, and K562 cells. Furthermore, the mechanistic analyses indicated that fangchinoline derivative **4g** could induce WM9 cell death by apoptotic means through suppression of the Bcl-2 family and activation of the caspase family with PARP. The present results suggest that compound **4g** could be a lead for development of anti-WM9 agents in the future.

## Figures and Tables

**Table 1:** The IC_50_ values of fangchinoline derivatives **1**–**4** against the growth of 5 cancer cell lines.

Compound	IC_50_ (µM)				
	HEL	PC3	WM9	K562	MDA-MB-231
**1a**	4.42	3.65	3.44	4.75	5.13
**1b**	4.01	7.22	5.56	6.60	4.44
**1c**	4.72	4.75	3.05	6.78	5.04
**2a**	8.96	3.69	3.04	8.33	8.60
**3a**	2.75	3.01	2.65	6.99	6.67
**3b**	2.95	3.59	3.95	3.85	4.61
**3c**	6.82	4.76	3.58	4.06	8.70
**3d**	2.04	4.39	3.05	3.35	1.99
**3e**	2.98	4.19	2.21	4.29	5.53
**3f**	3.29	4.29	2.61	4.32	4.08
**3g**	4.04	4.33	3.13	6.39	6.02
**3h**	1.86	2.51	4.57	3.62	2.79
**3i**	2.04	4.57	2.73	2.86	3.46
**4a**	2.65	4.22	2.51	2.45	4.12
**4b**	5.11	3.85	3.54	3.16	7.22
**4c**	3.52	2.58	2.48	3.83	3.96
**4d**	3.45	2.40	3.45	3.53	3.96
**4e**	4.08	3.83	3.60	5.27	4.16
**4f**	4.17	3.13	2.12	3.69	3.93
**4g**	1.98	2.03	1.07	2.09	2.92
Fangchinoline	8.95	11.06	13.13	10.29	12.29
Vincristine	6.38	5.36	10.59	5.62	13.14

From MTT assay after 48 h of treatment; the values are averaged from at least 3 independent experiments; variation ±10%.
